# Editorial: Obesity and type 2 diabetes mellitus: novel and alternative functional bioactive nutritional interventions

**DOI:** 10.3389/fendo.2024.1453733

**Published:** 2024-10-17

**Authors:** Renato Chaves Souto Branco, Luiz Felipe Barella, Isabella Lovizutto Iessi, Júlio Cezar de Oliveira

**Affiliations:** ^1^ Center for Diabetes and Metabolic Diseases, Indiana University School of Medicine, Indianapolis, IN, United States; ^2^ Gastrointestinal & Metabolism, Kallyope Inc., New York, NY, United States; ^3^ Lilly Diabetes Center of Excellence, Indiana Biosciences Research Institute, Indianapolis, IN, United States; ^4^ Institute of Health Sciences, Federal University of Mato Grosso, Sinop, MT, Brazil

**Keywords:** obesity, type 2 diabetes, bioactive compounds, nutritional intervention, molecular metabolism

Obesity is a metabolic disorder that increases the risk of developing type 2 diabetes mellitus (T2DM), cardiovascular disease and cancer and requires effective, durable interventions (1). Whilst dietary and physical activity approaches are foundational, long-term adherence is challenging. Pharmaceutical and surgical therapies are proven options but often unaffordable. Therefore, innovative and economically viable nutritional interventions, focusing on bioactive compounds, nutrients, or molecules are urgently required to address these disparities in adherence and access to effective therapies. This collection of articles focuses on novel dietary supplements and bioactive molecules extracted from functional food as strategies to mitigate or fight obesity and T2DM.

Bioactive nutritional components show promise in addressing obesity and associated complications (2). Chen et al. discusses the potential of traditional Chinese medicine, including acupuncture and herbal therapy, for improving metabolic health by regulating various metabolic processes such as energy homeostasis, glucose metabolism, inflammation and enhancing insulin sensitivity. Similarly, Zhou et al. demonstrated that mulberry leaf extract and *Hippophae* protein peptides (MHP) have beneficial effects on blood glucose, lipid profile, and weight loss. The administration of MHP in obese rats, suggests its potential for reducing adiposity through pathways like PPARγ and FGFR1 signaling, although further research is needed for a complete understanding of its mechanisms. The authors demonstrated MHP induced weight loss and reduced adiposity through blocking adipocyte enlargement and fat depots, despite similar energy intake in rats on high-fat high-fructose diet. Combined data supports the findings that the protective effect of MHP on adiposity is at least partially associated with PPARγ and FGFR1 signaling pathways.

As showed by Yang et al., *Faecalibacterium prausnitzii* strains, a potential bioactive-compound producing species, displays beneficial metabolic effects contributing to the amelioration of obesity and associated metabolic disorders in an obesity-mice model induced by high-fat diet consumption. In their study, the authors showed that *F. prausnitzii* acts by modulating the gut-brain axis, inducing gut and neural hormone secretion that inhibited appetite in rats. Similarly, a randomized double-blind controlled trial by Savytska et al., assessed live multi-strain probiotics combined with absorbent smectite supplement effectiveness in participants with T2DM. It showed improvement in glucose homeostasis and in the pancreatic β-cell function.

Metabolic dysregulation in obesity and T2DM is associated with overall body metabolic dysfunction, which involves the action of different hormones including incretins. Glucagon-like peptide 1 (GLP-1) receptor agonists are applicable for use in combination with metformin in selected clinical settings: atherosclerotic cardiovascular disease, T2DM, and the presence of chronic kidney disease (3, 4). Xie et al. aimed to perform a network meta-analysis to evaluate the efficacy and safety of GLP-1RAs in combination with metformin. This review provides evidence-based support and reference for the selection of clinical treatment. More specifically, the data showed that GLP-1RAs are highly effective in lowering HbA1c and reducing body weight and did not cause hypoglycemic reactions. Finally, the results presented here may provide guidance and orientation for the selection of clinical therapeutic agents.

Early intervention and well established anti-diabetic therapies are accepted and shown to be pivotal in the fight against obesity and its complications. However, sex-specific effects on endocrine/metabolism responsiveness are often observed and must be taken into consideration. Herein, Ivic et al. studied the effects of long-term treatment with metformin and liraglutide in elderly male and female rats fed a high-fat high-sugar. Even though, metformin treatment appears to be better than liraglutide, by improving central-leptin and peripheral-insulin sensitivity in female rats; while liraglutide therapy display a positive response, as short but not as long-term effect in male-rats, on the satiety signaling and on the hyperinsulinemia.
